# High dose rate ^192^Ir brachytherapy source model Monte Carlo dosimetry: mHDR-v2 and mHDR-v2r

**DOI:** 10.1371/journal.pone.0298550

**Published:** 2024-02-09

**Authors:** Shuhei Tsuji, Naomasa Narihiro, Masataka Oita, Yoshihito Namito, Hideo Hirayama

**Affiliations:** 1 Natural Sciences, Kawasaki Medical School, Kurashiki, Japan; 2 Department of Radiological Technology Faculty of Health Science and Technology, Kawasaki University of Medical Welfare, Kurashiki, Japan; 3 Department of Graduate School of Interdisciplinary Sciences and Engineering in Health Systems, Okayama University, Kita-ku Okayama, Japan; 4 High Energy Accelerator Research Organization, KEK, Tsukuba, Japan; Central State University, UNITED STATES

## Abstract

After 2010, the source model of the microSelectron HDR Afterloader System was slightly modified from the previous model. Granero et al. named the modified source model “mHDR-v2r (revised model mHDR-v2)” and the previous model “mHDR-v2”. They concluded that the dosimetric differences arising from the dimensional changes between the mHDR-v2 and mHDR-v2r designs were negligible at almost all locations (within 0.5% for *r* ≥ 0.25 cm), the two-dimensional anisotropy function difference between the two sources is found 2.1% at *r* = 1.0 cm when compared with the results of the other experimental group. To confirm this difference, we performed a full Monte Carlo simulation without energy-fluence approximation. This is useful near the radiation source where charged-particle equilibrium does not hold. The two-dimensional anisotropy function of the TG-43U1 dataset showed a few percent difference between the mHDR-v2r and mHDR-v2 sources. There was no agreement in the immediate vicinity of the source (0.10 cm and 0.25 cm), when compared to Granero et al. in mHDR-v2r sources. The differences in these two-dimensional anisotropy functions were identified.

## Introduction

Brachytherapy is a type of radiation therapy that is beneficial in the treatment of various cancers as it allows for a higher radiation dose to be directed at tumors and lowers the external radiation exposure to the surrounding tissues. Compared to external irradiation, brachytherapy has a steep dose distribution near the source, which allows for effective localized treatment of the tumor compared to external irradiation. Conventional brachytherapy treatments include a high dose rate (HDR) treatment, which involves delivering a high dose of radiation directly to the tumor through seed implantation. In brachytherapy, treatment plans are determined before initiating treatment. The radiation dose for HDR treatment is determined according to the TG-43U1 protocol [1]. Therefore, the source-specific data used in this protocol are critical for determining the exposure dose. Iridium (^192^Ir) is a typical source of radiation used in HDR brachytherapy. Since the actual dose in the vicinity of the radiation source is difficult to calculate, Monte Carlo (MC) simulations are performed to investigate the properties of the ^192^Ir source. Numerous studies have been conducted to clinically validate the TG-43U1 protocol for determining source models using ^192^Ir sources [1–14]. One such ^192^Ir source is the microSelectron HDR afterloader system model (Nucletron, Elekta AB, Stockholm, Sweden), which has been modified slightly since it was first reported by Daskalov et al. [7] This modified version was named “mHDR-v2r (revised model mHDR-v2)”, whereas the previous model was referred to as “mHDR-v2” by Granero et al. [9]. mHDR-v2 is no longer manufactured. Conversely, mHDR-v2r is currently widely used and is now referred to as mHDR-v2. Thus, for proper distinction, “mHDR-v2r (revised model mHDR-v2)” in the study by Granero et al. is hereafter referred to as “mHDR-v2r”, while “mHDR-v2” in the same study by Granero et al. is hereafter referred to as “mHDR-v2”. The HDR ^192^Ir brachytherapy sources of mHDR-v2 and mHDR-v2r were compared by Granero et al. using PENELOPE2008 MC simulation, including source electrons for dosimetry near the source. Granero et al. concluded that the dosimetric differences arising from dimensional changes between the mHDR-v2 and mHDR-v2r designs were negligible at almost all locations in the vicinity of the source, and a comparison of these results with prior MC studies typically showed agreement within 0.5% for *r* ≥ 0.25 cm.

**Fig 1:**
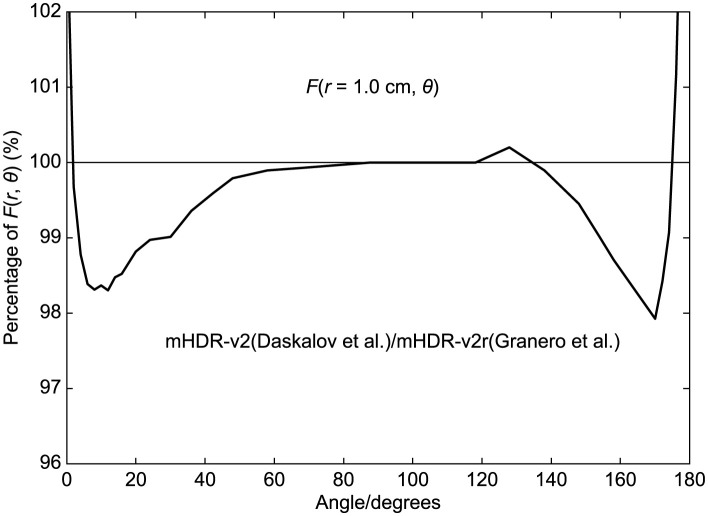
Ratio of 2D anisotropy function of the mHDR-v2 source (Daskalov et al.) to the mHDR-v2r source (Granero et al.) at 1.0 cm.

Fig 1 illustrates the comparison of two-dimensional (2D) anisotropy functions at 1.0 cm for mHDR-v2r, as investigated by Granero et al., and mHDR-v2, as studied by Daskalov et al. The ratio of $F_{v2}/F_{v2r}$ is presented as a percentage, where Granero et al.’s 2D anisotropy functions are denoted as $F_{v2r}$ and Daskalov’s as $F_{v2}$. Notably, the mHDR-v2 value was 1.7% lower at 12° and 2.1% lower at 170° compared to mHDR-v2r. This discrepancy contradicts Granero et al.’s previous conclusion of a difference of less than 0.5%. Currently, mHDR-v2 is not available, but if Granero et al.’s conclusions are to be accepted, it is possible to use mHDR-v2 data in treatment planning and deliver with mHDR-v2r. In this case, the patient will be exposed to 2% more radiation than planned. Therefore, it is crucial to assess the dosimetric differences between mHDR-v2 and mHDR-v2r sources. This study aimed to examine the mHDR-v2 and mHDR-v2r sources more comprehensively and assess the dosimetric differences between these two sources.

In this work, MC simulations were used to investigate the dosimetry differences between mHDR-v2 and mHDR-v2r. The simulation code was implemented using the Electron-Gamma Shower 5 (EGS5) code system [15]. For accurate irradiation planning in the immediate vicinity of the tumor, it is clinically important to include the influence of source electrons in the MC simulations. Generally, if the charged-particle equilibrium is upheld, the collision kerma and absorbed dose are considered equal. In addition, collision kerma can be converted from the energy fluence and mass-energy absorption coefficients. However, in order to accurately investigate the behavior of photons and electrons in the extreme vicinity, this MC simulation does not use approximations using energy fluences and mass-energy absorption coefficients. The absorbed dose or air kerma rate summarizes the energy deposit, or kinetic energy, of each particle emitted from a source. We evaluate the dosimetry differences between the two sources and present more detailed dosimetry results that are clinically useful. These findings are crucial for creating more accurate treatment plans in brachytherapy applications and improving patient safety.

## Materials and methods

### EGS5 code

EGS5 (Electron-Gamma Shower Version 5) code system employs a dual random hinge approach, effectively decoupling energy loss and multiple elastic scattering for modeling the spatial transport of electrons and positrons [15]. The primary advantages of this technique lie in that the random multiple scattering hinge preserves near second-order spatial moments of the transport equation over long step lengths. Additionally, the hinge mechanics are formulated to facilitate transport across boundaries between regions of differing media. In the EGS5 MC simulation, regions (called tallies in other MC simulation codes) were defined using combinatorial geometry (CG) subprograms. PEGS (Preprocessor for EGS) is a set of subprograms built into EGS5 that generates material data for use in the EGS5 code. To execute the EGS5 program, it is necessary to prepare data indicating the shape and location of the region (tally) and material data for PEGS that fills the region. A subroutine SHOWER program has been prepared, wherein the particle’s charge (0: photon, -1: electron, +1: positron), total energy, location, direction, region, and weight are described. The particle continues its trajectory until it reaches outside the region or falls below the cutoff energy. In specific scenarios, such as when the particle is transported over a distance (0), its energy is below the cutoff (1 and 2), the user requests its discard (3), or part of its energy is deposited due to binding energy (4), the subroutine AUSGAB program is called by default. When activated, the AUSGAB program aggregates the physical quantities possessed by particles. The step size in the program can be controlled by the “characteristic dimension” (CHARD). Generally, it is sufficient to set the smallest length of the region.

### Description of source materials

**Fig 2:**
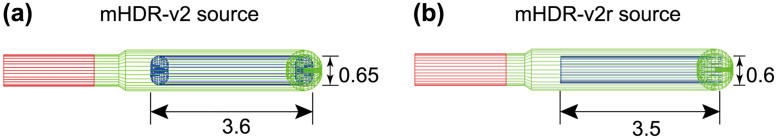
Schematic designs of ^192^Ir sources. Dimensions are given in mm. (a) mHDR-v2 source. Shapes and dimensions are according to Daskalov et al. [7](Fig 1(b)). (b) mHDR-v2r source. Shapes and dimensions are according to Granero et al. [9] (Fig 1).

The shapes of mHDR-v2 and mHDR-v2r are illustrated in Fig 2. The dimensions of the mHDR-v2 source used in the simulation were obtained from the studies by Daskalov et al. [7] (Fig 1(b)). The dimensions of the mHDR-v2r source were obtained from the study by Granero et al. [9] (Fig 1). As shown in Fig 2, the length and diameter of the Ir source of mHDR-v2r are 0.1 mm and 0.05 mm shorter than those of mHDR-v2. The radius of curvature of the head of the source capsules and the edge of the iridium (Ir) source of the mHDR-v2 model were accurately reproduced and described in detail in the EGS5 CG subprogram. According to the previous studies by Daskalov et al. and Granero et al., the composition weight ratios of the capsule and cable were 2% Mn, 1% Si, 17% Cr, 12% Ni, and 68% Fe. The length of the source cable was 2 mm. The densities of the capsules and cables were set to 8.02 gcm^-3^ and 4.81 gcm^-3^, respectively. In the mHDR-v2r model, the gap between the Ir source and capsule was dry air (0% humidity).

### Photon and electron spectra

**Fig 3:**
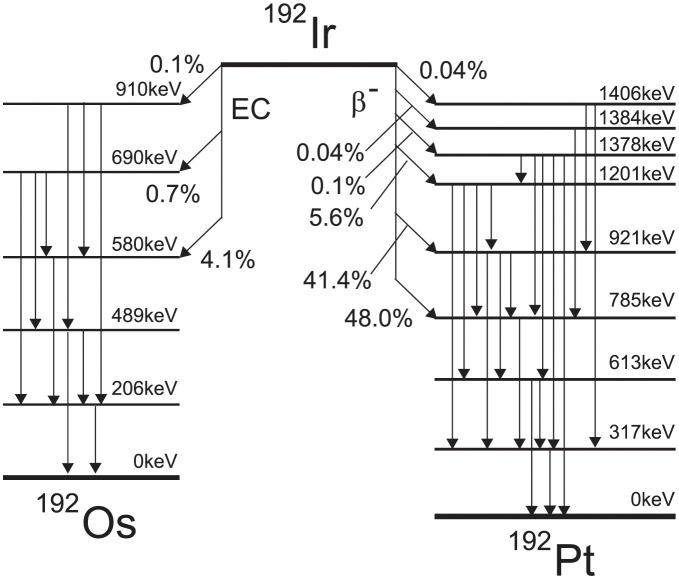
^192^Ir decay scheme.

As shown in Fig 3, ^192^Ir decays into ^192^Os and ^192^Pt. At this time, not only photons but also electrons are emitted. According to the decay scheme in Fig 3, the only electrons emitted are *β* decays, but contain internal conversion electrons and Auger electrons emitted by orbital electrons. The ^192^Ir photon spectrum produced by the National Nuclear Data Center [16, 17], which was subsequently quoted by Rivard et al. [8], was input into the MC simulations (total photon spectra = 2.2992 photons/Bq, with a cutoff energy of 10 keV). The electron spectrum included *β* decay, and internal conversion electrons were also used in the MC simulation, as recommended by the International Commission on Radiological Protection [18, 19]. The sum of the continuous spectra of *β* decay with a cutoff energy 10 keV was 0.9192 electrons/Bq (energy bin width × differential spectrum), whereas that of the internal conversion electrons with a cutoff energy of 10 keV was 0.1531 electrons/Bq. Auger electrons are excluded from consideration due to their energies being below 10 keV in the reference [18, 19]. The total summation was 1.0723 electrons/Bq. This value differs from that obtained by Granero et al. (1.113 electrons/Bq). The total of our electron spectra is 3.7% lower than that of Granero et al. We exclude source electrons with energies surpassing 669 keV. Granero et al. employed the electronic spectra, addressing internal conversion electrons above 700 keV and including Auger electrons in their analysis. In contrast, our approach did not consider Auger electrons, and these differences are considered a contributing factor to the observed lower electronic spectrum in our methodology.

### Coordinate system of MC and description of the detected volume

**Fig 4:**
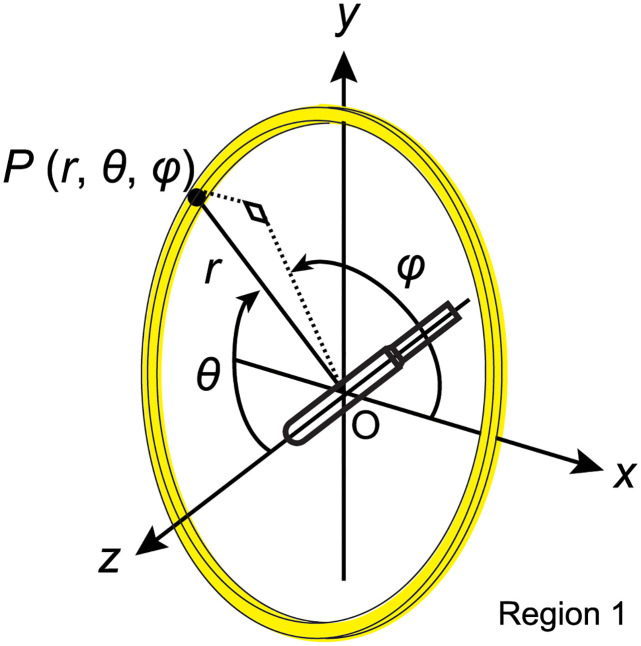
MC coordinate system. *r*, *θ* and *φ* of *P* are assumed, as shown in the figure. The data acquisition area is shown in yellow.

As shown in Fig 4, the coordinates define the longitudinal axis of the radiation source along the z-axis, with the tip direction of the radiation source from the cable considered positive. The center of the source is the origin of the coordinates. In the polar coordinate system, *r*, *θ* and *φ* represent the radius, polar angle, and azimuthal angle of the point of interest, *P*, respectively. The volume *V*(*P*) of *P*(*r*, *θ*, *φ*) sliced area was aggregated from *r* − Δ*r* to *r* + Δ*r* and *θ* − Δ*θ* to *θ* + Δ*θ*. This sliced area is highlighted in yellow in Fig 4 and represents the detected volume of *P*. The volume delineated in yellow is expressed by the following Eqs (1) and (2).

$$\begin{array}{ccc} V(P) & = & \int_{r-\Delta r}^{r+\Delta r}\int_{\theta -\Delta \theta }^{\theta +\Delta \theta }\int_{0}^{2\pi }r\text{sin}\left(\theta \right)d\varphi \cdot rd\theta \cdot dr \end{array}$$ (1)

$$\begin{array}{ccc} & = & \frac{2\pi }{3}\{{\left(r+\Delta r\right)}^{3}-{\left(r-\Delta r\right)}^{3}\}\{\text{cos}(\theta -\Delta \theta )-\text{cos}(\theta +\Delta \theta )\} \end{array}$$ (2)

### MC methods of dose rate

The hardware used for the MC simulations consisted of two computer clusters [20] connected by dozens of single-board computers and two personal computers with 8- and 4-core central processing units. All computers performed the calculations in parallel using the message-passing interface (MPI) technique. The simulation code used was MPI for EGS5, that is, EGS5MPI [21].

The following parameters were adopted in various regions or media: sampling of the angular distributions of photoelectrons, K- and L-edge fluorescent photons, K and L Auger electrons, Rayleigh scattering, linearly polarized photon scattering, incoherent scattering, and Doppler broadening of the Compton scattering energies. The transport cutoff energy (denoting the kinetic energies for electrons) level was defined as 10 keV for both electrons and photons. The MC simulation concludes tracking when the energy falls below the cutoff, and the remaining kinetic energy below the cutoff is deposited in the stopped area.

The photons and electrons were independently simulated as particles from the radiation source with cutoff energies of 10 keV. The diameter of the water-sphere phantom was set to 40 cm. Pure water, without gas, was used for the phantom, as recommended by TG-43U1 [1], at a density of 0.998 gcm^-3^. The part of the water-sphere phantom excluding the whole radiation source (^192^Ir, capsule and wire) is set as “Region 1” (tally in other MC simulations) (see Fig 4). Initially, particles (photons or electrons) are emitted randomly from within the volume of the radiation source enclosed within the capsule. The position is set randomly, along with a random direction, and energy is randomly selected by sampling from the energy spectrum, which is then fed into the SHOWER subroutine and executed. Upon calling the subroutine AUSGAB, the particle’s position (*x*, *y*, *z*) in Region 1 is provided. Subsequently, using the coordinates ($r=\sqrt{x^{2}+y^{2}+z^{2}}$, *θ* = arcsin(*z*/*r*)), it is determined whether particles fall within the target ranges (|*r* − *r*′| < Δ*r* and |*θ* − *θ*′| < Δ*θ*, *r*′, *θ*′:median target values). The energy deposit of the particle is aggregated in case within the target area. The total energy deposit is then divided by the small mass (volume of yellow area in Fig 4 × Region 1 density) to calculate the absorbed dose *D*. The absorbed dose rate, $\dot{D}$, is calculated for any *D* by considering the number of source photons and electrons, along with the emission rate of the radiation source. The *D* of *P* was tabulated from 0.05 to 20 cm. The aggregation width, Δ*r*, was 0.0025 cm from 0.05 to 1.5 cm and 0.025 cm from 2 to 20 cm. The angles were set from 0° to 180° at increments of 1°. For 0° < *θ* < 180°, the aggregation width was Δ*θ* = 0.1° (0.001745 rad), whereas that for *θ* = 0° or 180° was Δ*θ* = 0.5° (0.08727 rad) and only that data was aggregated from *θ* to *θ* ± Δ*θ*. The CHARD of Region 1, which controls the step size of the MC simulation, was set to 0.005 (cm). We calculated 1.0 × 10^11^ events for photons and electrons for each source, such that the standard error of the absorbed dose at *r* = 1 cm and *θ* = 90° was 0.2%.

### MC methods of air kerma strength

To obtain the air kerma, *K*(*r*), for the *r* of transverse-axis distances, the kinetic energy, which was transferred to the electrons by photons in a small region of the target air, was aggregated using the MC simulation and divided by the volume mass of that region. The air kerma rate, $\dot{K}\left(r\right)$, was calculated for *K*(*r*) by considering the number of photons and emission rate of the radiation source. The air kerma strength, *S*_*K*_, was calculated using a linear function fit from $\dot{K}\left(r\right)\cdot r^{2}$ for each *r*. Spherical shell phantoms of various radii were used to calculate *K*. These radii were located at 10 cm intervals (from 10 to 120 cm) from the center of the source. The thickness of the air layers was 1 mm, and the remaining space was a vacuum. For each *r*, the aggregation width was Δ*r* = 0.05 cm and Δ*θ* = 1° (0.01745 rad) at *θ* = 90°. The CHARD of the target area was set to 0.005 (cm) as well as the dose rate. The air was dry (0% humidity), as recommended by the AAPM 229 Report [10]. The weight compositionratios were N = 75.5%, O = 23.2%, and Ar = 1.3%. The density was 0.00120 gcm^-3^. Only photons with a cutoff energy of 10 keV were generated from the source. The number of events was 1.0 × 10^12^, such that the standard error was ≤0.3% at *r* = 10 cm.

## Results

We compared our results with those obtained by Granero et al. and other experimental groups. Granero et al.’s findings correspond to the mHDR-v2r source, while the results from other experimental groups pertain to the mHDR-v2 source. The cutoff energy of Granero et al.’s MC simulation was 10 keV for both electrons and photons.

### Contribution of source electron and gamma of each source

**Fig 5:**
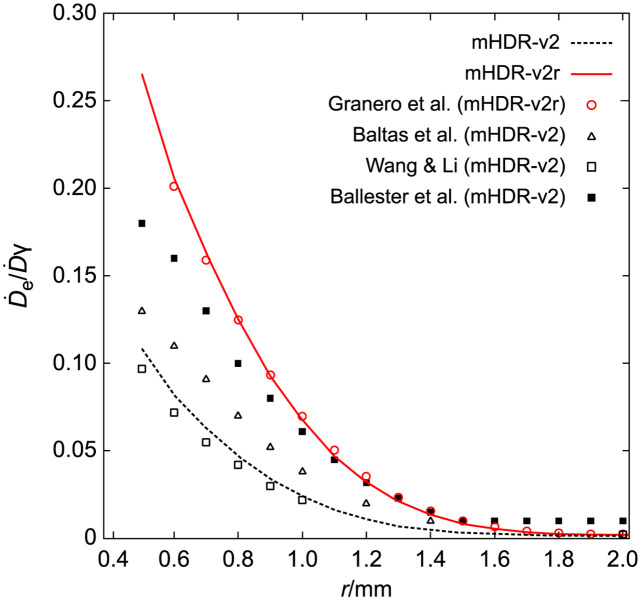
The ratio of absorbed dose source electrons to that of source photons near the radiation source.

Fig 5 illustrates the ratio of absorbed dose from source electrons to that from source photons in close proximity to the radiation source, alongside comparable results from other experimental groups [9, 11–13]. Granero et al.’s findings deviated from those of other experimental groups; however, our mHDR-v2r results are in line with those of Granero et al. Other groups utilized a source different from that of Granero et al.’s and our study, namely the mHDR-v2 type. The primary cause for this disparity lies in the design variation between mHDR-v2 and mHDR-v2r sources.

When comparing mHDR-v2 sources, our results were close to Wang & Li’s findings but diverged from those of other experimental groups using mHDR-v2. The discrepancies in results among various experimental groups employing the mHDR-v2 source can be attributed, in part, to the variation in the electron spectrum selection across studies [13].

**Fig 6:**
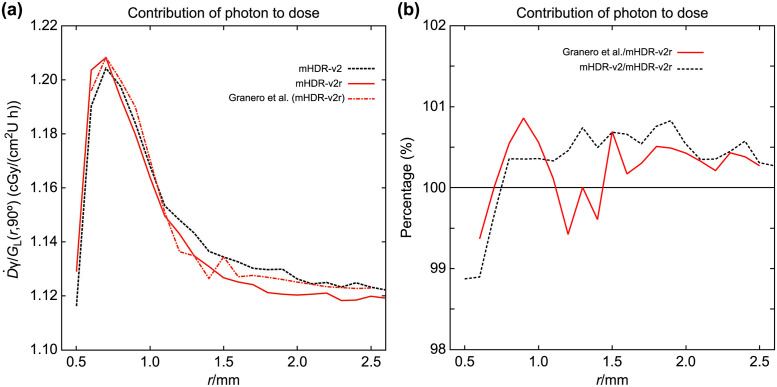
(a) Absorbed dose rate contributed by source photons near the source. (b) The ratio of the absorbed dose rate to mHDR-v2r source contributed by source photons near the source. The absorbed dose rate is normalized by the unit of air kerma strength and the geometry function.

**Fig 7:**
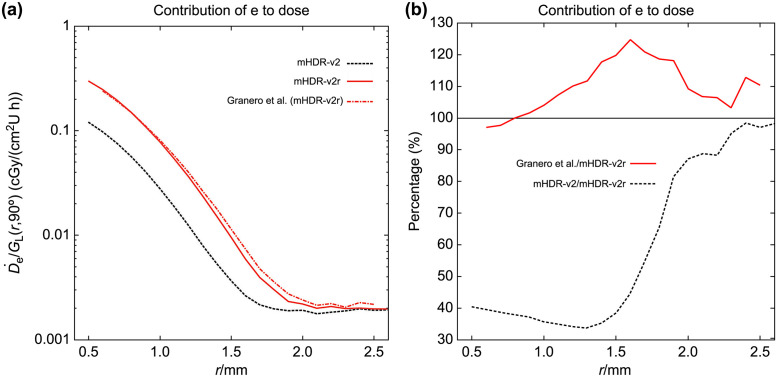
(a) Absorbed dose rate contributed by source electrons near the source. (b) The ratio of the absorbed dose rate to mHDR-v2r source contributed by source electrons near the source. The absorbed dose rate is normalized by the unit of air kerma strength and the geometry function.

In the assessment of our mHDR-v2 source, mHDR-v2r source, and the outcomes of Granero et al., we compared the contributions of the source electrons and photons toward the dose rates near the source. These contributions, along with the results of Granero et al., are presented in Figs 6 and 7. In Fig 6, the results for source photons in our mHDR-v2r and mHDR-v2 sources, as well as those of Granero et al. exhibit fluctuations within the 1% range, indicating consistency. Turning to Fig 7(a), the results for source electrons in our mHDR-v2r source appear consistent with those of Granero et al.; however, Fig 7(b) reveals a difference of ≥20% around 1.5 mm. This significant variance is attributed to the treatment of the electronic spectrum and the region where charged-particle equilibrium is not maintained. Specifically, our treated electronic spectrum was 3.7% lower than theirs. Despite this pronounced difference at 1.5 mm, the absorbed dose of electrons was less than 1% of that of photons, rendering it inconspicuous in Fig 5. Furthermore, the outcomes of our mHDR-v2 source deviate from those of our mHDR-v2r source, up to a distance of 2 mm, with a difference of >60%. This seems to be due to the difference in structure.

### TG-43U1 dataset: Dose rate constants for the model mHDR-v2r and mHDR-v2 sources

The air kerma strength, *S*_*K*_ (per 1 s and 1 Bq), obtained from the measurement of the air kerma rate, $\dot{K}$, at 12 points from 10 to 120 cm of the mHDR-v2r source was (2.7303 ± 0.0056) × 10^−11^ *μ*Gym^2^(Bqs)^−1^. The absorbed dose rate at *r* = 1 cm and *θ* = 90°, which is the dose rate constant Λ for the mHDR-v2r source, can be calculated as follows:

$$\begin{array}{c} \Lambda =\left(1.1186\pm 0.0031\right){\text{cGyh}}^{-1}U^{-1}\;\;\;\left(\text{mHDRv}2r\right) \end{array}$$

Similarly, the mHDR-v2 dose rate constant, Λ, can be calculated as follows:

$$\begin{array}{c} \Lambda =\left(1.1151\pm 0.0026\right){\text{cGyh}}^{-1}U^{-1}\;\;\;\left(\text{mHDRv}2\right) \end{array}$$

The value of the dose-rate constant for the mHDR-v2r source was 0.3% higher than that for the mHDR-v2 source. Granero et al. reported a value of (1.1121 ± 0.0008)cGyh^−1^U^−1^ for the mHDR-v2r source, whereas the consensus value for the mHDR-v2 source is (1.109 ± 0.012)cGy^−1^U^−1^ [10]. Our observations were slightly higher than the consensus value for the mHDR-v2 source but were within the margin of error.

### TG-43U1 dataset: Radial dose function for the model mHDR-v2 and mHDR-v2r source

**Fig 8:**
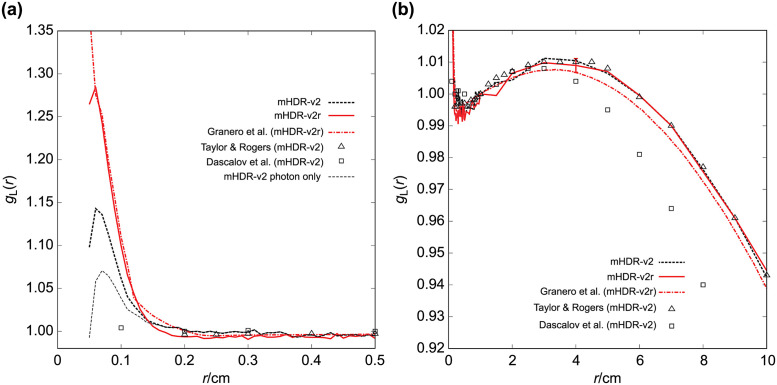
Comparison of the radial dose functions. (a) 0 cm ≤ *r* ≤ 0.5 cm. (b) 0 cm ≤ *r* ≤ 10 cm. The standard error is added at *r* = 4 cm.

**Fig 9:**
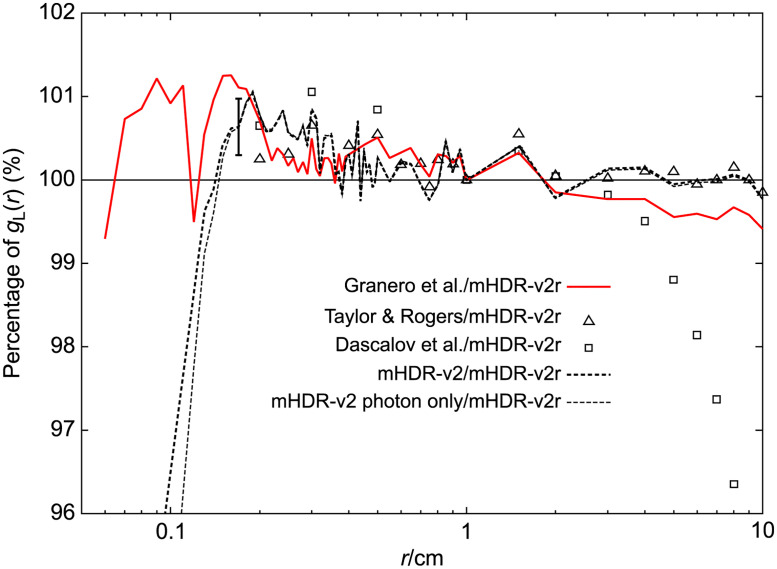
The ratio of the radial dose functions to the mHDR-v2r source.

The results of the radial dose function *g*_*L*_(*r*) are shown in Fig 8, along with those of the other experimental groups [7, 9, 14]. According to Granero et al., the vicinity of the mHDR-v2r source exhibited a sharp peak, which is inconsistent with the observations of other experimental results. Our mHDR-v2r results were consistent with those of Granero et al. (Fig 8(a)). A comparison of the mHDR-v2 and mHDR-v2r sources revealed a difference depending on the vicinity. In addition, considering only the photons emitted from the mHDR-v2 source exhibited a slight peak in *g*_*L*_(*r*), which differed from the results of Daskalov et al. The ratio of each result to the mHDR-v2r of *g*_*L*_(*r*) is shown in Fig 9. The difference between our results and those of Granero et al. was >1% in some places (<0.2 cm), but it was generally <1%. Our results and those of the other experiments were consistent within 1.1% (0.2–4 cm).

### TG-43U1 dataset: 2D anisotropy function for the model mHDR-v2 and mHDR-v2r sources

**Fig 10:**
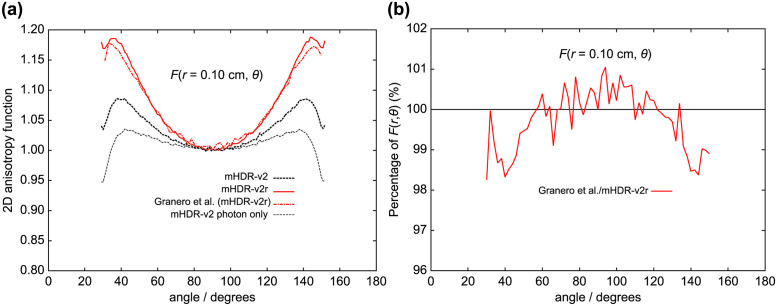
(a) Comparison of 2D anisotropy functions at distances of 0.10 cm. (b) The ratio of 2D anisotropy functions to mHDR-v2r source at 0.10 cm.

**Fig 11:**
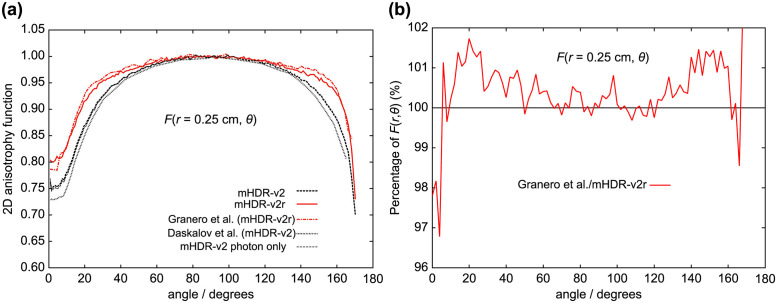
(a) Comparison of 2D anisotropy functions at distances of 0.25 cm. (b) The ratio of 2D anisotropy functions to mHDR-v2r source at 0.25 cm.

**Fig 12:**
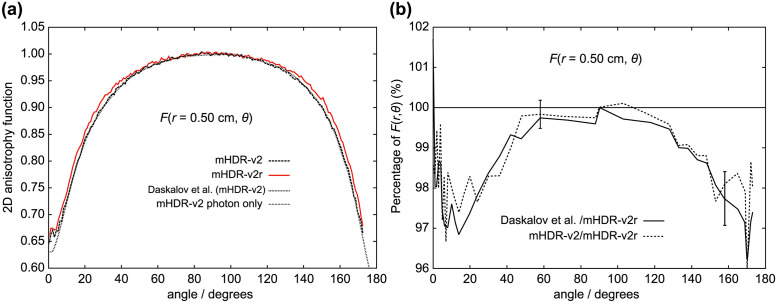
(a) Comparison of 2D anisotropy functions at distances of 0.50 cm. (b) The ratio of 2D anisotropy functions to mHDR-v2r source at 0.50 cm. The error bar at 58° is derived from the relationship between mHDR-v2r and mHDR-v2, while the error bar at 158° is derived from the relationship between mHDR-v2r and Daskalov et al.

**Fig 13:**
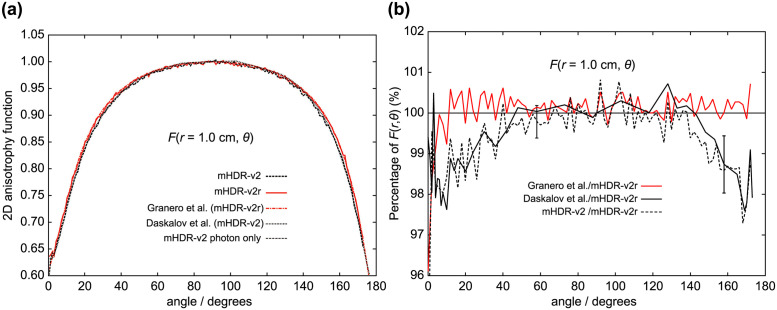
(a) Comparison of 2D anisotropy functions at distances of 1.0 cm. (b) The ratio of 2D anisotropy functions to mHDR-v2r source at 1.0 cm. The error bar at 58° is derived from the relationship between mHDR-v2r and mHDR-v2, while the error bar at 158° is derived from the relationship between mHDR-v2r and Daskalov et al.

The results of the 2D anisotropy function, *F*(*r*, *θ*), are shown in Figs 10–13, along with those of a previous experimental group [7, 9]. In each figure, (a) shows the 2D anisotropy function value, and (b) shows the ratio of the 2D anisotropy function in each experiment to mHDR-v2r. From Fig 10 (*r* = 0.10 cm), the results of Granero et al. and those of our mHDR-v2r source appear to be consistent. However, the results of Granero et al. deviate from our results by −1.7% at 40°, + 1.1% at 94°, and −1.6% at 144°.

From Fig 11 (*r* = 0.25 cm), the results of Granero et al. deviate from ours by + 1.7% at 20° and + 1.5% at 144°. From Fig 12 (*r* = 0.50 cm), the difference between the mHDR-v2r and mHDR-v2 sources was −2.6% at 14°, −4.2% at 170° and the results of mHDR-v2r are inconsistent with the results of mHDR-v2 and Daskalov et al. However, the results of mHDR-v2 and Daskalov et al. are in good agreement. From Fig 13 (*r* = 1.0 cm), the results for the mHDR-v2r source were consistent with those of Granero et al. The differences between the mHDR-v2r and mHDR-v2 sources were −2.0% at 8° and−2.2% at 170°. The error bars in Figs 12 and 13 are for mHDR-v2r and mHDR-v2 at 58° and for mHDR-v2r and Daskalov et al. at 158°, respectively. These *F*(*r*, *θ*) differences are given in % in Table 1.

**Table 1:** The difference of *F*(*r*, *θ*) given in %.

	*F*(*r* = 0.10cm)			*F*(*r* = 0.25cm)		*F*(*r* = 0.5cm)		*F*(*r* = 1.0cm)		
*θ*	40°	94°	144°	20°	144°	14°	170°	8°	12°	170°
Granero et al./mHDR-v2r	−1.7	+ 1.1	−1.6	+ 1.7	+ 1.5	-	-	-	-	-
mHDR-v2/mHDR-v2r	-	-	-	-	-	−2.6	−4.2	−2.0	-	−2.2
Daskalov.et al./Granero et al.	-	-	-	-	-	-	-	-	−1.7	−2.1

### Uncertainties of typical $\dot{D}\left(0.10\text{cm},90^{\circ }\right)$, $\dot{D}\left(1.0\text{cm},90^{\circ }\right)$, and $\dot{K}\left(10\text{cm}\right)$

For the uncertainty of the source geometry, capsule geometry, dynamic source design, ^192^Ir photon spectrum, and MC physics, please refer to the studies by Granero et al. [9] and Rivard et al. [8]. The uncertainty of the ^192^Ir electron spectrum is 0.03% from nuclear decay data for dosimetry calculation (DECDC2) [19]. The uncertainty of volume averaging represents the difference between the median extrapolated from the volume-averaged distance and the value aggregated in the bin. The uncertainties of volume averaging at $\dot{D}(1.0$ cm, 90°) and $\dot{K}(10$ cm) were <0.002%. The total statistics depended on the number of events aggregated in the bin. There was almost no difference between the mHDR-v2r and mHDR-v2 sources. The uncertainties are presented in Table 2. The total uncertainties of $\dot{D}(0.10$ cm, 90°), $\dot{D}(1.0$ cm, 90°), and $\dot{K}(10$ cm) were 4.15%, 1.19%, and 1.14%, respectively. When the comparison was performed using MC simulation, the uncertainties of the source geometry, capsule geometry, and dynamic source design generated in the manufacturing process were excluded. In this case, the uncertainties of $\dot{D}\left(0.10\text{cm},90^{\circ }\right)$, $\dot{D}\left(1.0\text{cm},90^{\circ }\right)$, and $\dot{K}\left(10\text{cm}\right)$ were 1.01%, 1.02%, and 1.05%, respectively.

**Table 2:** Uncertainty analysis for the mHDR-v2 and mHDR-v2r ^192^Ir brachytherapy sources based on MC simulations. Types A and B uncertainty components are categorized based on stochastic and systematic effects, respectively.

	$\dot{D}\left(0.10\text{cm},90^{\circ }\right)$		$\dot{D}\left(1.0\text{cm},90^{\circ }\right)$		$\dot{K}\left(10\text{cm}\right)$	
Component	Type A	Type B	Type A	Type B	Type A	Type B
Source geometry	-	0.46%	-	0.46%	-	0.46%
Capsule geometry	-	0.01%	-	0.01%	-	0.01%
Dynamic source design	-	4%	-	0.4%	-	0.04%
^192^Ir photon spectrum	-	1%	-	1%	-	1%
^192^Ir electron spectrum	-	0.03%	-	0.03%	-	0.03%
MC physics	-	0.05%	-	0.05%	-	0.05%
Tally volume averaging	-	0.03%	-	-	-	-
Tally statistics	0.14%	-	0.19%	-	0.30%	-
Total (k = 1) uncertainty	4.15%		1.19%		1.14%	

## Discussion

### Applicability of results

We performed dosimetry of the mHDR-v2r and mHDR-v2 sources using full MC simulation. In MC simulations, the difference in the results may be attributed to the differences in the radiological physics models used in the coding systems. Figs 12 and 13 show the overwhelming agreement between the results of Daskalov et al. and our mHDR-v2 source results. Daskalov et al.ï¿½fs study uses similar radiological physics models, although the MC simulation code is different and warrants the validity of the EGS5 simulation code. We also investigated the contribution of source photons and/or source electrons to the mHDR-v2r and mHDR-v2 sources. A difference of more than 60% in the absorbed dose rate of the source electrons between the mHDR-v2r and mHDR-v2 sources was found. Granero et al. reported that the difference between mHDR-v2 and mHDR-v2r within 0.25 cm is attributed to the dosimetric contribution from source electrons and the absence of electronic equilibrium but not the difference in the source design. If the difference in the source design mentioned by Granero et al. is negligible, the black-dotted and red solid lines shown in Figs 5 and 7 should overlap when comparing only our results. This implies that the design difference between mHDR-v2r and mHDR-v2 in the vicinity of the source cannot be ignored. Further, Fig 13 in the 2D anisotropy function shows that: (i) our results were consistent with the results of Granero et al. when comparing mHDR-v2r sources at a distance of 1 cm, (ii) our results were consistent with the results of Daskalov et al., when comparing mHDR-v2 sources at a distance of 1 cm, (iii) from (i) and (ii), the 2D anisotropy functions of the mHDR-v2r and mHDR-v2 sources do not match even at a distance of 1 cm. These results are consistent with the difference in the 2D anisotropy function at 1 cm between mHDR-v2r (the results of Granero et al.) and mHDR-v2 (the results of Daskalov et al.) mentioned earlier (Fig 1). This is entirely due to the design differences between the mHDR-v2 and mHDR-v2r sources. Therefore, the effect of design differences between the mHDR-v2r and mHDR-v2 sources cannot be ignored even at a distance of 1 cm from the source. In clinical practice, mHDR-v2 data should not be used for treatment planning when using the mHDR-v2r source. As a result, the patient risks receiving more than 2% of the radiation near the longitudinal direction of the source. A comparison between the results of Granero et al. and our mHDR-v2r source results also showed differences in the source electron contribution (Fig 7) and in the 2D anisotropy function within 0.25 cm (Figs 10 and 11). One reason for this difference seems to be the difference in treatment in the region where the charged-particle equilibrium does not hold. As mentioned above, our MC simulation aggregates the energy deposit or kinetic energy for each particle emitted from the source. Although there are many source particles in the MC simulation to obtain statistical data, this method makes it irrelevant whether it is in the region of charged-particle equilibrium. At clinical sites, polyamide implant tubes with an outer diameter of 1.7 mm are prepared for brachytherapy. This is required for treatment remarkably close to the source. Due to the commercialization of such tubes, accurate treatment planning in the vicinity of the radiation source is required. Our results are also especially useful in such clinical settings.

### Statistical data and error margins

From Table 2, “Dynamic source design” occupies the largest proportion of the total uncertainty of $\dot{D}\left(0.10\text{cm},90^{\circ }\right)$. This results from lateral shifts in the manufacturing process of the source. The maximum lateral displacement is estimated to be 0.02 mm [9]. Hence, this value means “maximum” rather than standard error. Excluding this, the uncertainties are around 1%. The 1% uncertainty is also due to the “^192^Ir photon spectrum”. If the equivalent photon spectrum is used, the comparison of results between MC simulations is performed with sufficiently high accuracy and can be used as basic data in clinical practice. However, in clinical practice, whether or not brachytherapy in the vicinity of the radiation source can be performed with sufficient accuracy depends on the accuracy of the radiation source manufacturing process.

### MC simulations and limitations

Granero et al. published the first dosimetry results including electrons. This is commendable in the MC simulation of brachytherapy. Similar to Granero et al., we conducted comprehensive MC simulations to contain the source electrons. These simulations refrain from using approximations involving energy fluences and mass-energy absorption coefficients, because it is important not to use approximations to obtain accurate results regardless of whether the charged-particle equilibrium holds. If MC simulations are performed and analyzed, approximations should not be used, despite the lengthy time requirements for such simulations. The time it takes to incorporate various elements and perform detailed simulations is a limitation of MC simulation. To make the simulations more realistic, it is necessary to incorporate various elements into MC simulations.

## Conclusions

The absorbed dose rate and air kerma rate of the mHDR-v2r and mHDR-v2 sources were examined using EGS5 with full MC simulations. Comparison of the mHDR-v2r and mHDR-v2 sources revealed that the difference in the absorbed dose was caused by the electron and structure. The TG-43U1 dataset was obtained by MC simulation. For the dose-rate constant values, both mHDR-v2r and mHDR-v2 were greater than those reported by Granero et al., and the consensus values were within the margin of error. For the radial dose function, the mHDR-v2r source and those of Granero et al. were generally consistent, within 1%, while the mHDR-v2r and mHDR-v2 sources were consistent at distances >0.2 mm. A comparison of the mHDR-v2r and mHDR-v2 sources for the 2D anisotropy function revealed a difference of −4.2% at 0.50 cm and a difference of −2.2% at 1.0 cm. These differences indicate that when using the mHDR-v2r source, its accuracy is not sufficient to use the TG-43U1 dataset of the previous mHDR-v2 source, even at distances greater than 0.5 cm. Also, for the mHDR-v2r source, there was a difference of −1.7% to + 1.1% at a distance of 0.10 cm and a difference of + 1.7% at a distance of 0.25 cm when compared with that of Granero et al. Thus, the results of Granero et al. and our mHDR-v2r sources are inconsistent. The results of this study may provide valuable insight into the treatment planning of HDR brachytherapy.

## Supporting information

S1 Data Dose data for mHDR-v2 and mHDR-v2r. Data supporting the findings of this study are available in the supplementary material of this article. The supplementary material contains detailed dosimetric data, such as source structures, absorbed dose rates, TG-43U1 datasets, and air kerma rates associated with the dose rate constants. The source structure is written in CG so that it can be used with EGS5. The following supporting information can be downloaded at:www.xxx.xxx/xxx/s1, Supplementary Data: Dose data for mHDR-v2 and mHDR-v2r.
(XLSX)
